# Smart urban governance: an alternative to technocratic “smartness”

**DOI:** 10.1007/s10708-020-10326-w

**Published:** 2020-11-09

**Authors:** Huaxiong Jiang, Stan Geertman, Patrick Witte

**Affiliations:** Faculty of Geosciences, Department of Human Geography and Spatial Planning, Utrecht University, Utrecht, 3584 CB The Netherlands

**Keywords:** Smart cities, Urban challenges, Smart governance, ICT, Contextualization

## Abstract

This paper argues for a specific urban planning perspective on smart governance that we call “smart urban governance,” which represents a move away from the technocratic way of governing cities often found in smart cities. A framework on smart urban governance is proposed on the basis of three intertwined key components, namely spatial, institutional, and technological components. To test the applicability of the framework, we conducted an international questionnaire survey on smart city projects. We then identified and discursively analyzed two smart city projects—Smart Nation Singapore and Helsinki Smart City—to illustrate how this framework works in practice. The questionnaire survey revealed that smart urban governance varies remarkably: As urban issues differ in different contexts, the governance modes and relevant ICT functionalities applied also differ considerably. Moreover, the case analysis indicates that a focus on substantive urban challenges helps to define appropriate modes of governance and develop dedicated technologies that can contribute to solving specific smart city challenges. The analyses of both cases highlight the importance of context (cultural, political, economic, etc.) in analyzing interactions between the components. In this, smart urban governance promotes a sociotechnical way of governing cities in the “smart” era by starting with the urban issue at stake, promoting demand-driven governance modes, and shaping technological intelligence more socially, given the specific context.

## Introduction

The pressure of urbanization coupled with lingering economic instability and global climate change has created various new challenges for cities, such as traffic congestion, crime, economic stagnation, population segregation and air pollution (Batty et al. 2012; Hollands 2008). To deal with these urban challenges, the notion of the smart city has been proposed as a potential solution. In many countries, smart cities are developed to increase equitable access to basic urban services, such as education, healthcare, sanitation, drinking water, and mobility. Local governments expect that by employing various smart ICTs, operational and managerial efficiency, citizen engagement in service co-production, and quality of life can be significantly improved. Although the concept of the smart city is considered to have great potential, associated governance challenges have prevented cities from achieving the expected outcomes (Ruhlandt 2018). As Barns (2018, p. 6) comments, the ideals of the smart city in seeking to benefit from digital services necessitate a “*reinvention of governance.*”

The recent increase in research into the concept of smart governance is one such effort seeking to achieve the better governance of the smart city (Ruhlandt 2018; Webster and Leleux 2018; Scholl and AlAwadhi 2016; Scholl and Scholl 2014). Smart governance emerges mainly due to the growing role of technology in the functioning of cities, which has made governmental agencies rethink their roles in such data-rich cities (Bolívar and Meijer 2016). Smart governance can use various smart technologies (e.g., big data, Internet of Things (IoTs), and Artificial Intelligence (AI)) to upgrade traditional administrative systems (e.g., e-government) to the city level by streamlining city operations, making better decisions, and delivering improved quality of life (Pereira et al. 2018; Webster and Leleux 2018).

However, smart governance in practice is strongly characterized by a supply-oriented, technocratic way of governing cities (e.g., Marvin et al. 2015). In this process, much emphasis is put on the role of technology in collecting data and producing knowledge to smarten government operations and automate urban system functions (Jiang et al. 2020a, b; Verrest and Pfeffer 2019; Kitchin et al. 2016; Kitchin 2014). Such an approach focusing on digital and technology-driven innovation is often considered to be a universal solution to varied urban issues in different cities (Verrest and Pfeffer 2019). According to some authors, technocratic “smart” governance conceals those urban issues, conflicts, and controversies that cannot be represented by digital tools and data analytics (e.g., social discrimination and mental illness) (Bina et al. 2020; Cardullo and Kitchin 2019; Hashem et al. 2016; Rathore et al. 2016).

Therefore, many authors urge that more transformative and sociotechnical governance approaches are needed to transform the current form of smart governance (Jiang et al. 2019a, b; Dano et al. 2019; Joss et al. 2019; Webster and Leleux 2018; Ruhlandt 2018). For instance, Meijer and Bolívar (2016) argue that smart governance should promote new forms of human collaboration through the use of ICTs to obtain better outcomes and more open governance processes. For them, more emphasis should be put on social inclusion, social capital, and sustainability; thereafter, we should study smart governance as a complex process of institutional change and acknowledge the political nature of appealing visions of sociotechnical governance. Verrest and Pfeffer (2019, p. 1329) highlight that there is a failure to consider the “urban” as a response to “*what urban challenges related to smart cities are and what appropriate [governance] solutions are.*” This perspective indicates that we need to become more aware of how urban problems and their proposed smart solutions are socially constructed. In response to the calls for transformative “smart” governance, some authors argue that we must start with the “urban” and not with the “smart,” shifting from a technology-pushed to an application-pulled governance approach, and shaping technologies socially (Jiang et al. 2020a, b; Tomor et al. 2019; McFarlane and Söderström 2017; Stratigea et al. 2015).

Based on the above, the aim of this paper is to present a specific urban planning perspective on smart governance: smart urban governance. The contribution of smart urban governance moves away from technocratic smart governance toward promoting an urban social process of smart governance innovation. In this context, Jiang et al. (2019b, p. 247) stress that real “smart” governance should integrate “the ‘smart’ from smart governance literature” with “the ‘urban’ from urban governance literature,” as a means of “smartening” urban governance and highlighting the importance of urban dynamics in shaping smart governance. This paper presents three interconnected components of smart urban governance, namely the spatial (substantive urban challenges), institutional (modes of governance), and technological components (technological intelligence). By examining them and showing how they interact with each other, mediated by context specificities, it proffers a context-based, sociotechnical response to urban challenges related to smart cities and opens up new possibilities for transformative city governance.

The remainder of this paper is organized as follows. Section 2 focuses on the theoretical background and evaluates the dominant perspective on the smart governance debates. The three abovementioned components are discussed in detail in Section 3 and a context-based, sociotechnical governance approach—smart urban governance—is framed to connect these components. Section 4 introduces the research methodology. Two sets of empirical analyses are presented in Section 5 to show the added value of the framework. Section 6 discusses the findings and their potential implications, and concludes this paper.

## Theoretical background

### Smart city: opportunities and challenges still coexist

It has been over 10 years since the smart city concept was explicitly advocated by Hollands (2008). In literature, there are two overarching approaches to discussing smart cities, namely the technology-driven approach and the human-driven approach. A recurring aspect in the definition of a smart city is the use of ICTs. According to the technology-driven approach, smart cities focus on the acceptance and use of technologies, and their integration into the city infrastructure, to increase efficiency and effectiveness in the city environment (Greenfield 2013; Batty et al. 2012). Accordingly, policymakers and ICT suppliers are expected to come together to plan smart cities and deploy ICT-based solutions (Cardullo and Kitchin 2019; Simonofski et al. 2019; Calzada and Cobo 2015; Shelton et al. 2015).

In contrast, the human-driven approach highlights that the use of ICTs by communities must enable them to participate more fully in so-called knowledge societies (Barns 2018; Jiang et al. 2020a; Leydesdorff and Deakin 2011). For instance, Neirotti et al. (2014) argue that smart cities should take advantage of the opportunities offered by ICT to involve multi-actor, multi-sector, and multilevel perspectives and promote community-based smart city building. Kummitha and Crutzen (2017) emphasize that smart cities need to create more avenues for social interactions between different stakeholders and enhance the skills and capabilities of local people and communities to benefit their daily life. In this perspective, smart cities should be seen from a user-centered view with more emphasis on citizens and other stakeholders than on the technology itself.

Based on the differing priorities within smart cities, Caragliu et al. (2011) stress that a comprehensive definition of the smart city concept is needed to incorporate the multiple strands. They consider a city as smart “*when investments in human and social capital and traditional (transport) and modern (ICT) communication infrastructure fuel sustainable economic growth and a high quality of life, with a wise management of natural resources, through participatory governance*” (Caragliu et al. 2011, p. 70). According to this definition, the concept of smart cities should promote people-centered development, incorporate ICTs into urban management, and stimulate the design of an effective government that includes collaborative planning and citizen participation.

In practice, however, the development of smart cities is over-reliant on the deployment of ICTs or technological infrastructures, and neglects social services of general interest (Monachesi 2020; Desdemoustier et al. 2019; Simonofski et al. 2019; Datta 2015). As a consequence, many smart city initiatives are criticized for their “*self-proclaiming and self-congratulatory*” notions of such smartness (Hollands 2008, p. 62). As noted by some scholars, the concept of smart city is simply used as a business model for large high-tech companies to market their technology products and to privatize public space (Kitchin et al. 2016; Marvin et al. 2015). It is seen by some authors as paving the way for a corporatization of city governance that largely excludes the interests and contributions of ordinary people (Shelton and Lodato 2019; Grossi and Pianezzi 2017).

The failure to recognize the value of bottom-up innovation has increased social inequality (Simonofski et al. 2019; Effing and Groot 2016). Although there is no doubt that ICTs can help create new knowledge and discover improved ways of governing cities, ICTs are just an enabler, not a panacea for all the problems and issues faced by cities and humankind (Joss et al. 2019; Kummitha and Crutzen 2017). Various services can be offered to citizens via ICT-augmented government systems, but not everyone in the city can benefit from those services, especially people with a low socioeconomic status and those who are marginalized or excluded in some way (e.g., refugees, migrants, asylum seekers) (Cardullo and Kitchin 2019; Simonofski et al. 2019; Willis 2017).

As Bolívar (2018, p. 1) asserts, “*many of the challenges to be faced by smart cities surpass the capacities, capabilities, and reaches of their traditional institutions and their classical processes of governing*.” For smart cities to be effective, there is a need to critically evaluate the present governance of smart cities and to promote more transformative governance approaches (Jiang et al. 2020b; Dano et al. 2019; Ruhlandt 2018).

### Smart governance: a critical review

As a component of smart cities (Caragliu et al. 2011), the smart governance concept is being increasingly employed by policymakers and private companies to create smarter cities by using key terms such as smart decision-making, smart administration, and smart collaboration (Ruhlandt 2018; Scholl and Scholl 2014). However, there is no commonly accepted definition of smart governance. Based on an extensive literature review, it seems that smart governance can mean (1) making the right policy choices (cf. Nam 2012), (2) developing innovative governance structures via ICT (cf. Meijer and Bolívar 2016), or (3) governing with a focus on the outcome, that is, dealing with substantive urban challenges (cf. Jiang et al. 2019b). Elaborating on the concept of smart governance from these three angles adds to a better understanding of the concept.

In practice, many authors have demonstrated the added value of smart governance for smartening a city and promoting a high quality of life. For instance, Scholl and AlAwadhi (2016) show that ICT-enabled governance facilitates collaboration between different cities to provide smart services that no single municipality can provide alone. Meijer and Thaens (2018) assert that smart governance supports the collection of data to strengthen the governance of urban safety. More recently, smart governance has been used to handle the COVID-19 pandemic in South Korea by facilitating proactive information-sharing and enabling citizens to understand the situation and follow the newly released safety guidelines (Choi et al. 2020).

Although smart governance shows great potential for “smart” city developments, smart governance has been criticized for its technocratic way of governing cities (Jiang et al. 2020a, b; Verrest and Pfeffer 2019; Barns 2018). In this process, governments treat the smart governance of cities merely as a management issue that can be dealt with by making use of the power of data analytics (Krivý 2018; Shelton et al. 2015; Kitchin 2014). In practice, several examples can be found of decision-makers in government that perceive important urban problems as being solvable primarily through the application of technologically derived knowledge; for instance, by transforming the characteristics of local places (geology and landform) and human-related variables (gender and religion) into configurable report tables and graphs (Hashem et al. 2016; Rathore et al. 2016). The assumption underlying this technocratic approach is that knowledge produced with the help of technology is considered “value-free” and “objective,” and will unbiasedly help governance. Furthermore, due to the failure to consider the urban setting, the place-based knowledge of local people can hardly be received and reflected in the formulation and production of policy content (Bina et al. 2020; Cardullo and Kitchin 2019; Söderström et al. 2014). In short, technocratic smart governance neglects the role of contextualization in shaping the governance process.

In addition, the implementation of smart governance is often closely related to the ideological nature of the discourse around neoliberalism, implying its close association with corporate interests (Jiang et al. 2020a; Sadowski 2020; Barns 2018; Hollands 2015). According to Springer et al. (2016), neoliberalism in practice is usually aligned with policies of economic liberalization, such as privatization, lowering taxes, free trade, and reductions in government spending and regulations. As for urban governance and urban development, neoliberalism implies making the public sector more efficient through processes of marketization and the outsourcing of urban services to private companies (Jessop 2002). As many smart city initiatives show, ideas about urban development are often closely related to the imaginations and plans of key private corporations (e.g., IBM’s Smarter Planet and Cisco’s Smart+Connected Communities) (Wiig 2015). Governments then play an active role in facilitating the process of designing, creating, and implementing policies for smart city development (Hollands 2015). As Luque-Ayala et al. (2016) note, the implementation of smart governance helps private corporations to sell their “smart” packages and local governments to promote their political and social interests. However, the interests of local people are usually largely excluded from such governance processes (Jiang et al. 2020a; McFarlane and Söderström 2017). Consequently, smart governance in practice typically presents a situation in which power, wealth, and business capital play a key role in directing and controlling the discourses and practices of smart cities (Krivý 2018; Kitchin 2014).

Furthermore, in some countries—for example, China, Vietnam, and Saudi Arabia—technocratic smart governance controversially enhances the authoritarian and potentially oppressive systems of governance (Keegan 2020; Anderlini 2019; Fountain 2001; Pali and Schuilenburg 2019). For instance, in China the governance-oriented City Brain project in Hangzhou employs advanced video monitoring, facial recognition systems, and predictive policing to monitor, anticipate, and influence the behavior of individuals and certain groups (Beall 2018; The Trend Letter 2017). Although it significantly enhances the governing capabilities of Hangzhou city government, according to some authors, the networks and techniques of surveillance and control largely acted as generators of feelings of discomfort and uneasiness in citizens (Beall 2018) and consequently reduces their mental health and wellbeing (Whittaker 2019; Pali and Schuilenburg 2019). Similarly, in other projects like Songdo Ubiquitous City, South Korea, and Masdar City in the United Arab Emirates, actions taken by governments, businesses, and other organizations as a result of big data analytics produce privacy and security concerns (Kuecker and Hartley 2020; Angelidou 2017).

Thus, rather than offering innovative and effective approaches for dealing with various urban problems, the shortfalls of present-day smart governance have created extra challenges for smart city developments. Several authors argue that smart governance has focused too much on the technical, engineering, and economic dimensions, while there is a lack of consideration for the role of urban social processes in shaping and configuring its meaning in practice (Faraji et al. 2019; Krivý 2018; Marvin et al. 2015; Söderström et al. 2014). Smart governance largely leaves the smart to the powerful (government and corporate elites) rather than foregrounding smart in the lifeworld of different stakeholders (especially citizens) in the city (Datta 2015). The “place-based, experiential” knowledge generated through the wishes, demands, requirements, and conditions of ordinary people—especially the urban poor and the marginalized—is often ignored (McFarlane and Söderström 2017, p. 318). In addition, the technocratic way of governing cities can hardly take into account the ways in which residents learn what really matters in their urban environment and how that might be supported. The outcome of technocratic smart governance may be highly unequal in urban societies, characterized by unequal power relations, social exclusion, and unbalanced distributions of costs and benefits (Kitchin et al. 2016). Therefore, for transformative smart governance, we must better understand the reasons for the acceptance or rejection of a technology as an appropriate solution for specific urban problems (Jiang et al. 2020a, b; Tomor et al. 2019; Verrest and Pfeffer 2019; Joss et al. 2019; Ruhlandt 2018).

## Smart urban governance: three interrelated components

In line with the foregoing, in this section we further elaborate upon smart urban governance by identifying its three key components—namely its spatial, institutional, and technological components—and their interrelationships.

### Spatial component: urban challenges

When smart governance is concerned with urban space, it considers this foremost as the spatial carrier of governance objects (Jiang et al. 2019b). However, from a smart urban governance perspective, the urban space constitutes the diversity of urban challenges that ask for governance action. It should be noted that urban studies have a long tradition of critically examining the interface between urban challenges and digital technologies (Graham and Marvin 2002). For instance, the introduction of a technological innovation often originates from handling urgent urban challenges like mobility congestion or social segregation issues (Vonk 2006). Consequently, in smart urban governance, narratives and practices around the notion of smartness should focus not merely on the problem-solving powers of big data, city sensors, and intelligent infrastructure, but primarily on the role of urban challenges in stipulating the functional support of technological innovations (Jiang et al. 2020b). In that, a prime focus on the pressing urban challenges can enhance the capabilities of ICT to contribute to the problem-solving nature of the governance object.

**Fig. 1:**
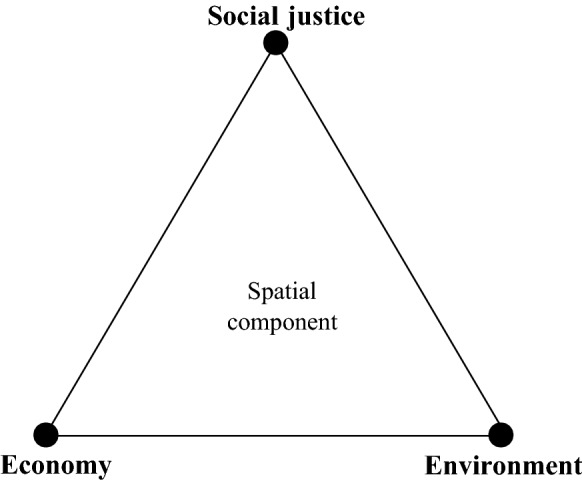
Spatial component—urban challenges (based on Campbell 1996)

In accordance with the concept of “sustainability,” Fig. 1 illustrates the associated main urban challenges, namely “to grow the economy, distribute the growth fairly, and in the process not degrade the ecosystem” (Campbell 1996, p. 3). It points out the main urban challenges faced by contemporary cities and indicates the targets that smart rationalities and techniques should meet. In particular, the trade-offs between the sustainability goals can be considered a huge urban challenge. As such, we believe that the model of economic, social, and ecological claims and the trade-offs between them to arrive at “sustainability” is in itself of value to frame the nature of urban challenges; it thus constitutes the “spatial” component of our concept of smart urban governance.

### Institutional component: modes of governance

Smart urban governance also needs input and contributions from various groups and organizations. To successfully deal with pressing urban challenges, actors from the state, market, and/or civil society have to collaborate in innovative ways, or “modes of governance” (Driessen et al. 2012). This differs sharply from the notion of technocratic smart governance, which emphasizes either the government as the prime initiator of innovative solutions, or the private sector as the provider of ICT-based smart solutions.

**Fig. 2:**
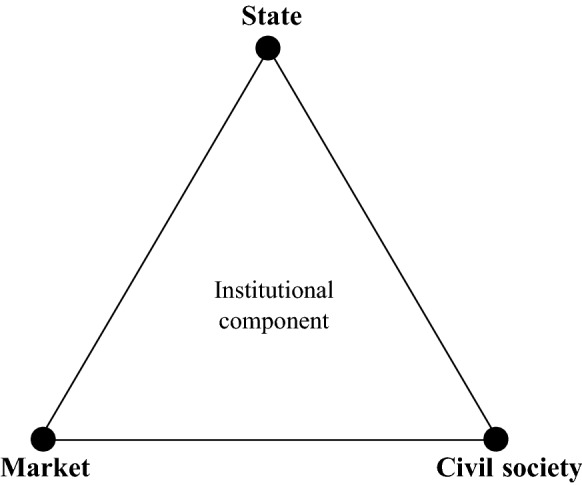
Institutional component: modes of governance

The literature discusses distinct structures of governance. However, each mode of governance implies the involvement, in some form, of the three mentioned types of actors (Driessen et al. 2012). Based on the degree of power sharing between these actors in the decision-making process, the structure of governance can be classified as either authoritative, competitive, or cooperative (Roberts 2000). Figure 2 integrates the abovementioned actors and their collaboration, which constitutes the institutional component of smart urban governance. The basic idea of this triangle is that the institutional component within smart urban governance is composed of the interactions between actors from the state, market, and/or civil society to arrive at well-intended solutions.

### Technological component: functional intelligence

The previous subsections show that smart urban governance should start from urban challenges and be attuned to the wider group of involved urban actors. As for the role of technology in smart urban governance, it means that technological innovation should satisfy the real needs of the actors within governance practices to be able to deal with pressing urban challenges (Jiang et al. 2019a, 2020a, b; Meijer and Thaens 2018).

**Fig. 3:**
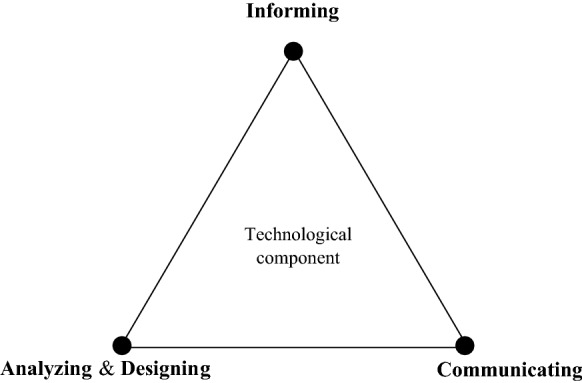
Technological component: functional intelligence

In technological innovation studies, each technological artifact has different meanings and interpretations for various actors. Thus, smart urban governance should build upon the knowledge, ideas, and opinions of different actors to create innovative technological functions that can satisfy their real needs. To do so, in smart urban governance the technological component is envisioned by its functional intelligence. Based on Geertman (2014) and Vonk (2006), these information-handling capabilities of technologies can be categorized into three groups: “informing ICT,” “communicating ICT,” and “analyzing and designing ICT.” The first capability—informing ICT—is intended to make governance-related knowledge and information accessible and interpretable from an access point or sender toward a user. The second—communicating ICT—is aimed at facilitating communication and discussion processes between those involved in the governance process by supporting flows of information between them (Pelzer 2015). And the third capability—analyzing and designing ICT—is intended to facilitate the advanced processing of data to detect urban patterns and the underlying processes, in order to facilitate the perception, creation, and presentation of design ideas (Geertman 2014). These distinctive functional intelligences provide different urban actors with the proper support capabilities to deal with the diversity of urban challenges. For instance, the communicating capability of ICT can help build collaborative forms of decision-making, while the analyzing capability of ICT can help users to process data and facilitate the simulation of potential solutions to urban problems. The functional intelligence represents the “technological” component of smart urban governance and is illustrated in Fig. 3.

### A context-based, sociotechnical governance approach

**Fig. 4:**
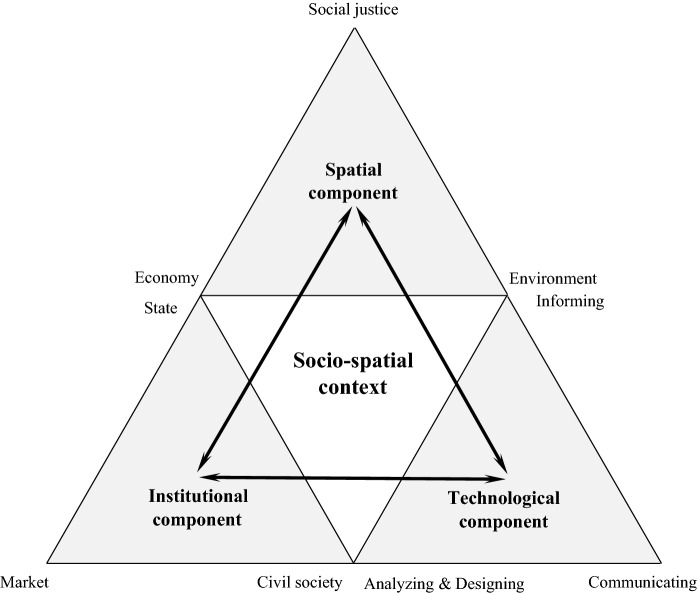
Smart urban governance framework

The three abovementioned components can be integrated into a conceptual framework for smart urban governance (Fig. 4). This framework indicates how the three interrelated components can achieve a balanced governance structure. The three thicker arrows show the interrelationships between the spatial, institutional, and technological components. The figure thus represents a state of co-evolution whereby one component interacts closely with the others and in which changes in one component will have consequences for the others. These interactions are crucial to avoid the previously mentioned technocratic way of governing cities and form the sociotechnical response to smartening city governance.

In addition, smart urban governance can only function properly when it is put into the specific socio-spatial context of a city (Geertman 2006; Jiang et al. 2019b). According to Jiang et al. (2020a), five key contextual factors can be identified from the smart city governance literature, that is, economic, political, cultural, and technological factors and the urban issue itself. Unlike previous smart governance approaches, this smart urban governance framework underlines the importance of these local urban contextual characteristics that should therefore be explicitly taken into consideration.

Smart urban governance strives to create a context-focused, sociotechnical governance approach to coordinate and steer the objectives, actors, and artifacts, namely urban challenges, institutional modes of governance, and technological intelligence. It stresses that smart urban governance departs from the urban challenges (=goal) and from that identifies the appropriate modes of governance and technologies (=means), given the context in which it is embedded. Thus, the smartness of smart urban governance refers to the potential of its components’ interactions, in a specific context, to increase our capacity to handle urban challenges, enhance stakeholders’ capabilities for collaboration, and improve technology’s usefulness, all aimed at achieving smart city development. In the following sections, with the help of an international questionnaire survey and two illustrative cases collected via index systems, we demonstrate the added value of this framework in practice.

## Methodology

We first discuss how we conducted the questionnaire survey, which was used to show the applicability of the smart urban governance framework. We then explain how we selected the two illustrative cases to demonstrate the detailed working mechanisms of the framework.

### Survey on smart city projects worldwide

In May–July 2019, we sent a questionnaire to the Computers in Urban Planning and Urban Management (CUPUM) research community via electronic and regular mailing lists. About 1300 people worldwide were invited to fill in the questionnaire. The reason for selecting the CUPUM community as respondents was that: (1) CUPUM is a major international academic conference that provides an advanced platform for the dissemination of information and knowledge on the science and technology of big data, smart cities, and smart urban futures (Geertman et al. 2019); and (2) participants of CUPUM (mainly scholars, technologists, and doctoral students) possess comprehensive knowledge and skills and rich experiences related to ICT application in city governance and planning. Thus, they offered a highly professional overview of smart urban governance in the context of smart cities. Of the approximately 1300 questionnaires sent out, 268 were completed by respondents (response rate of just over 20%). Of these completed questionnaires, 175 had been filled out by respondents who had been professionally involved in smart city projects. We therefore used their questionnaires in our analysis.

The questionnaire had two parts. The first part gathered basic data on the (anonymous) participants, such as gender, age, profession, origin, and expertise in the use of ICT. Respondents were also asked about their expertise and personal experiences with smart city projects. The second part gathered in-depth information about the different features of the framework (e.g., context, urban problems, governance modes, types of technologies) for smart urban governance in practice.^1^ We carried out statistical analysis of the statements relevant to this study to demonstrate the applicability of the smart urban governance framework in a wide variety of smart city cases.

### Stepping into two illustrative cases

Using the data obtained through the questionnaire, we focus on two smart city projects—Smart Nation Singapore and Helsinki Smart City—to illustrate the detailed working mechanisms of the framework. The selection of these two case studies was based on an extensive review of key international literature and of smart city projects worldwide.^2^ Two sets of data—policy documents and data related to smart city practices—were gathered and studied to examine the governance processes of the two cases. First, online search engines were used to collect policy-related documents based on keywords (e.g., “Singapore Smart Nation” and “Helsinki Smart City”). A snowball sampling method enabled the tracking and collecting of other potentially relevant policy documents. Second, local government portals and academic search engines were used to gather data related to these smart city practices. The practice-related data were mainly derived from academic literature, governmental portals, social media blogs, and digital newspaper archives.

Discourse analysis—which reveals the meaning of texts and other forms of communication in their social and institutional contexts—was applied to investigate the various features and their significance for smart urban governance in both cases. The present research employed two key dimensions of discourse analysis developed by Fairclough (1995). First, the units of analysis of a text analysis are empirical evidence of the latent meaning found in the discourse analysis. Therefore, text analysis was used to determine the features of the smart urban governance framework. Second, social practice requires a study of discourses in relation to wider power structures and social and cultural contexts. Based on the discursive analysis of each case, we compared the similarities and differences between smart urban governance in these two projects.

### Analysis guidelines

Following the conceptual framework, the analysis (1) focused on the urgent urban issues facing cities; (2) examined how the characteristics of the urban issue influence or define the choice of a specific mode of governance; (3) explored how the urban issue and the selected governance mode together determine the choice of functional intelligence (ICT functionality); and (4) enquired into the role of contextual factors in mediating the interactions of the components of smart urban governance. Below we demonstrate how the smart urban governance framework can contribute to analyzing a context-focused, sociotechnical way of governing cities.

## Smart urban governance in practice

In this section, the results obtained via the questionnaire survey are presented to show the applicability of the smart urban governance framework in a wider range of smart city cases. This is followed by two illustrative smart city cases, which show the detailed working mechanisms of the framework.

### Applicability of smart urban governance in wider contexts

Concerning geographical origin, most of the respondents (53%) came from China; the others came from Europe (15.4%), Asia (excluding China) (14.2%), Oceania (5.1%), South America (5.1%), North America (5.1%), and Africa (2.3%). This indicates the variety of the socio-spatial contexts in which smart urban governance is embedded.

In terms of types of urban issues handled, the majority of issues (61.2%) were mixed urban issues (combinations of either economic, social, or environmental issues), while 24.6% of the projects were related to only economic issues, 8.5% to only social issues, and 5.7% to only environmental issues.

To handle these issues, various modes of governance were applied: 12.6% of the projects adopted a centralized mode of governance, 28% a decentralized mode of governance, 8% public–private governance, 44.6% an interactive mode of governance, and 6.9% self-governance. The frequency (absolute number) of use of each governance mode in handling the different types of urban issues (see Jiang et al. 2020c) shows that centralized and decentralized governance were mainly employed to solve economic issues (mostly transportation and mobility), while the other governance modes were typically used to solve mixed urban issues. No governance modes were created to exclusively handle either social (e.g., housing) or environmental issues.

Furthermore, in terms of types of ICT applied to support governance processes and handle urban issues, 2.8% of the projects used only informing ICT, 1.7% only communicating ICT, and 48% only analyzing and designing ICT; 47% adopted hybrid ICT tools (combinations of either informing, communicating or analyzing and designing ICT). We also calculated the frequency (absolute number) of the use of each type of ICT in supporting governance processes and handling urban issues (see Jiang et al. 2020c). First, concerning the linkages between ICT and governance processes, analyzing and designing ICT was mainly used to support decentralized and interactive governance modes, whereas informing ICT and communicating ICT were primarily applied to improve interactive governance modes; few ICT tools were adopted to support public–private governance and self-governance. Second, concerning the linkages between ICT and urban issues, analyzing and designing ICT was typically used to handle mixed urban issues, while informing ICT and communicating ICT were applied to handle economic issues (mainly transportation and mobility issues); few ICT tools were exclusively used to handle either social or environmental issues.

The questionnaire revealed that smart urban governance varies significantly in different socio-spatial contexts. As urban issues differ in different countries, the modes of governance and types of technologies applied also differ. This implies that smart urban governance contextualizes itself and forms a sociotechnical response to urban challenges in the context of smart cities. In the next subsections, we discuss two illustrative cases to show how this context-based, sociotechnical way of governing cities (smart urban governance) works in practice.

### Two illustrative cases

#### Smart Nation Singapore

##### *Urban issues*

In recent decades, Singapore’s main urban issues (high energy consumption; insufficient transportation infrastructure and solid waste management; inadequate housing; high unemployment; and environmental vulnerabilities) have been exacerbated by rapid urbanization, increasing urban density, and the high demands of urban environments. More recent changing structures of international competitiveness, along with Singapore’s increasing burdens of an ageing population, a widening income gap, and declining productivity, further magnify the negative impact on the city’s sustainable development (Bhaskaran 2018). Against this background, the Smart Nation project was launched by the government as a nationwide effort to take advantage of the recent emergence of smart ICTs (e.g., immersive media, AI, IoT, and robotics) to handle these sustainability challenges (Tan and Zhou 2018).

##### *Governance choice*

Influenced by Singapore’s massive urban issues, along with its top-down institutions and dominant government-led approaches (Ho 2017), the government adopted a “whole-of-government” centralized approach to govern the Smart Nation initiative at the national scale (Khern 2019). Two key government agencies—Smart Nation and Digital Government Group (SNDGG) and Government Technology Agency (GovTech)—placed under the Prime Minister’s Office (PMO) were established in 2017 as the central governing body for the Smart Nation initiative. The position of Singapore as a city-state with limited natural and social resources requires it to stimulate innovative advances (e.g., productivity improvement and knowledge economy) and create successful transitions to a more sustainable and resilient future (Cavada et al. 2019; Hoe 2016). As Chesbrough (2006) argues, the nature and characteristics of innovative activities call for the involvement of multiple stakeholders to jointly test, develop, and create smart solutions. Accordingly, the focal point of urban governance in Singapore has also witnessed the emergence of government-led participatory and collaborative approaches to solve its complex and intertwined urban problems (Tan and Zhou 2018).

##### *Selection of ICT functionality*

To support the whole-of-government approach and handle service-relevant issues, the abovementioned “informing” functionality was initially created and applied to facilitate the governing of the Smart Nation initiative. For instance, web-based ICTs were used to radically overhaul the city-state’s existing government systems and to build a comprehensive, digital government administration platform—Core Operations, Development Environment, and eXchange (CODEX)—to deal with the segmented e-citizen services and applications. A transformative open government data portal (data.gov.sg) was then launched to provide one-stop access to the government’s publicly available datasets, covering health, transportation, education, housing, the environment, etc. Various communicating ICTs such as online platforms and networks were also developed by government-linked companies to build a system of mechanisms for collaborative innovation. The best illustration of this government-led, ICT-enabled collaboration is the development of startup companies and innovations in technology-based services and products. For instance, AI Singapore—an online innovation platform aiming to engage all Singapore-based ecosystems of AI startups, AI producers, and research institutions—was established by a government-wide partnership comprising the SNDGG, National Research Foundation, Integrated Health Information Systems, etc.^3^ Through crowdsourcing, hackathons, and living labs, it supports new startup companies and/or develops technology-based solutions to address Singapore’s urban problems.

The government’s efforts in recent decades to improve Singaporeans’ digital literacy and technology skills have enabled ordinary people to utilize neighborhood forums, blogs, and websites to improve the way they live, work, and play (Cavada et al. 2019). For instance, a government-facilitated crowdsourcing portal “eCitizen Ideas” allows citizens to share or contribute their opinions, suggestions, and ideas related to daily issues faced by the public, often through campaigns, competitions, and hackathons organized by various government agencies (Woo 2017). Also, collaborations between elderly people and state-owned companies have facilitated the development of the Smart Elderly Alert System, which tracks the movement and activities of the elderly and enables them to live independently. In addition, social media like Twitter and Facebook are also used by innovation enthusiasts to engage in some of the aforementioned innovation activities (e.g., co-production of healthcare services), or curate events and host discussions around new technologies such as blockchains, MedTech, and IoT (Khern 2019).

##### *Role of contextual factors*

Looking at the interactions of urban issues, governance modes, and ICT functionality in Singapore, we also see the importance of the embedded context (e.g., political institutions, resource constraints, and technological basis) in analyzing the development, implementation, and effects of smart urban governance. For instance, influenced by Singapore’s massive urban issues and its top-down political tradition, a whole-of-government approach was initially applied to enhance the participatory efforts of various government agencies and enable data to be exploited across individual, organizational, and national boundaries. Then, the position of Singapore as a city-state with limited resources led to more collaborative approaches aimed at mobilizing the strength of the whole of Singapore to address its issues. However, due to the government’s special relationship with the consortium (i.e. government-linked companies), the government and its agent companies still have a role within the collaboration process (Cavada et al. 2019). Influenced by this, ICTs and web-based telecommunication technologies were used either to improve the government’s capabilities to deliver efficient and effective services, or to make use of the knowledge and insight of local people to boost urban innovations and improve residents’ quality of life. This thus reflects a combination of more state-led, informing intelligence and more collaborative governance approaches in Singapore.

#### Helsinki smart city

##### *Urban issues*

Helsinki’s rapid urbanization over the past 20 years has led to a range of urban issues that could restrict its ability to create a sustainable future. Population growth driven by migration has greatly increased the demand for public services, such as energy provision, transportation infrastructure, housing, and employment. In addition, localized environmental problems such as indoor air pollution, vehicle emissions, and the pollution of lakes and coastal areas threaten the living conditions of Helsinki’s residents. Against this background, in 2014 the Helsinki government initiated the Helsinki Smart City program to handle these sustainability challenges (Research and Innovation Strategy for Regional Development 2014–2020, updated by Helsinki Smart Region—Strategy Update 2018–2020 in 2017). As Laakso (2017) illustrates, the overall purpose of Helsinki’s transition toward a smarter city is to create new business models, improve residents’ quality of life, and make Helsinki more sustainable and resilient.

##### *Governance choice*

According to Anttiroiko (2016), Helsinki—Finland’s capital city—is characterized by its democratic tradition and bottom-up institutions and decision-making processes. Influenced by this, smart urban governance in Helsinki has been approached, since its inception, from an integrative perspective on urban problems, using triple helix collaborations (Hämäläinen 2020). An ideal illustration of Helsinki’s smart urban governance is the Smart Kalasatama project initiated by Helsinki City Council in 2013 to become a co-created model district for smart living (e.g., unique housing, accessible and flexible living, sports, recreation, greenery). Considering city residents both as the most precious resources and the beneficial owners, the Smart Kalasatama project itself acts as the test and experimentation environment for different stakeholders (mainly enterprises, urban planners, local citizens, and students) to co-create the district.

##### *Selection of ICT functionality*

To support Helsinki’s smart urban collaboration, practices showed that right from the beginning, the Helsinki government has used an integrative innovation platform—Forum Virium Helsinki—to co-produce the Helsinki Smart City with universities, companies, and local citizens. The platform serves a wide range of roles (e.g., innovation communities, growth services, participatory and collaborative urban design, and investment). Since its establishment in the mid-2000s, Forum Virium Helsinki has advanced and witnessed a booming growth of living labs, crowdsourcing, open data, urban services, and mobile apps. For instance, Helsinki Living Lab was established and is coordinated by Forum Virium Helsinki to engage interested groups and absorb their new ideas and innovative concepts for service innovation. By using distributed user interfaces on the spot or via the web-based platforms, interested groups can participate in various co-production and/or co-creation activities, such as healthy neighborhoods, mobile services tests, waste collection systems, and future schools.

The applied participatory and citizen-based governance not only enhanced the capabilities of Helsinki to provide functional services, but also fostered social responsibility for tackling urban issues that are of collective concern. Influenced by this, integrating digitally assisted tools with face-to-face interaction creates self-organized innovation spaces that allow local residents to collaborate at the same level as experts (researchers) to discuss and make community-based plans. An example of this is the Aalto Built Environment Lab.^4^ Facilitated by large projection displays and support equipment, such as microphones and cameras, planning experts from Aalto University work and communicate collaboratively with broader community stakeholders (e.g., city planners, politicians, residents, and landowners). By further using ICT-enabled data analytics and visualizations to present the issues of concern, discussions between engaged stakeholders co-produce a large variety of ideas, suggestions, and knowledge as the foundation for planning their community. According to Anttiroiko (2016), the governance of Helsinki Smart City is largely built on ICT-enabled, user-oriented “platformization” to mobilize public data and local knowledge and provide tailored services and solutions.

##### *Role of contextual factors*

Helsinki’s democratic culture and active civil society, along with its bottom-up decision-making process, have enabled the municipal government to tackle its sustainability challenges based on wider collaboration between governments, businesses, citizens, and research institutions. In such an environment, civic engagement and collaboration are often considered the key features of Helsinki’s smart city development. Many solutions to Helsinki’s urban challenges have been the result of community-based collaborations between citizens, businesses, and local government, rather than being produced in a top-down bureaucratic way. Various smart technologies (e.g., living labs, platforms, and service- and user-oriented apps) have been developed to engage different stakeholders, especially citizens, to participate in the co-production of services that meet their real needs. Consequently, smart urban governance in Helsinki shows how a people-centered issue (smart living) provides a co-innovative setting in which diverse stakeholders jointly test and create smart solutions through online and offline platforms (Anttiroiko 2016).

#### Comparison and reflection

The analysis first indicates that, as urban issues differ in Singapore and Helsinki, the appropriate governance modes and relevant ICT functionalities applied also differ. As mentioned, “smart nation” Singapore endeavors to handle both strategic issues that have a long-term impact on survival, and daily issues that influence the quality of life (Hoe 2016). Because of this, it adopted a combination of whole-of-government, centralized, and more collaborative approaches. As for the role of technological intelligence, informing and communicating ICTs are developed and implemented to either deliver public services or facilitate collaborative activities (e.g., product and service innovation). In contrast, “co-created smart” Helsinki shows more concern about the living environment and the level of wellbeing offered to its inhabitants. Therefore, more citizen-centric, integrated, and ICT-facilitated flat structures were selected to govern the Helsinki Smart City project. In terms of the role of technologies, integrative functionalities (informing, communicating, and/or analyzing and designing) allow decision-makers to derive valuable insights into issues, something that previously was not possible. In addition, these technologies greatly facilitate open innovation, experimentation, and citizen engagement in the co-creation and co-production of urban services and urban living.

Second, the analysis also shows the importance of the specific context (cultural, political, economic, etc.) in influencing the choices both within each component and in their interaction, resulting in distinct forms of smart urban governance. In Singapore, massive urban issues along with top-down institutions put the government at the center of efforts to develop and pilot government-led, informative platforms seeking smart solutions. The position of Singapore as a city-state with limited natural and social resources and its efforts to equip people with digital skills, have also created ICT-facilitated, city-wide collaborations with businesses, interested citizens, and knowledge institutes. Taken together, smart urban governance in Singapore indicates the nationwide and whole-of-government effort along with the increased state-citizen engagement to reshape Singapore’s policy processes and transform the living environments of Singaporeans (Hoe 2016). In Helsinki, influenced by Finland’s democratic tradition, innovation culture, and strong technological basis, triple helix collaborations and integrative innovation platforms were developed to handle major issues and problems confronting residents’ everyday lives. As a result, smart urban governance in Helsinki suggests an extended public-sector innovation, with technologically enabled platforms serving to enhance the reach and efficacy of co-creation and co-production mechanisms. According to Zhou (2017), context is vital since the environment in which a typical governance is embedded limits, confines, or shapes the development and implementation of that governance approach. Stakeholders should therefore understand that urban processes are always interlinked and intertwined, and that smart governance mechanisms ought to be contextualized and comprehended as compound, synthesized actions.

Third, the analysis shows that the smart urban governance framework (Fig. 4) provides an effective analytical method to decide how to govern cities in the smart era. Although Singapore and Helsinki are confronted with different urban issues and are embedded in different urban contexts, both have obtained positive outcomes and needed improvements in terms of economic development, e-government innovation, public service delivery, quality of life, etc. (Monachesi 2020; Calder 2016). The key to this is that by adopting a forward-looking and problem-oriented strategy, both highlight that the development of modes of governance and relevant ICT functionality should accord with the perceived economic, social, and/or environmental urban challenges. In addition, the framework explicitly proposes analyses of both the choice of each component and the interactions of the components in a larger urban context. By doing so, smart urban governance moves away from a simple technology-based policy intervention toward a more compound and contextualized comprehension of how interactions of the urban issues, urban actors, and urban technologies engage in generating smart solutions and of their impacts on contemporary urban life.

## Conclusions

We have argued for a specific urban planning perspective on smart governance (i.e., smart urban governance) that aims to overcome the deficiencies of the present technocratic way of governing smart cities. The smart urban governance framework departs from pressing urban challenges, selects appropriate modes of governance, and advocates the proper application of the technology to the problem at hand and the needs of users within the particularities of the socio-spatial context. Such a governance approach integrates technology into the urban setting and facilitates an interactive relation between the urban dynamics and technology-facilitated governance. It implies that the smartness of smart urban governance is not just derived from its power to implement and reconfigure technology, but also relies heavily on its ability to respond to the changing urban setting and create new sets of social relations.

The questionnaire on smart city projects worldwide revealed that smart urban governance varies remarkably in different socio-spatial contexts. As urban issues differ in different countries, the governance modes and relevant ICT functionalities being applied also differ. The case studies of Singapore and Helsinki showed that taking the urban challenges as a starting point helps to define appropriate governance structures and to develop dedicated technologies that contribute to the successful governance of these smart cities. In general, this asks for the close attunement of the particular spatial, institutional, and technological components to the context at hand. In terms of transforming the role of technology in current smart cities, it implies that the technology should be closely embedded in the appropriate mode of governance and be closely related to the substantive urban problems at hand. Both empirical analyses highlight the importance of the specific context (cultural, political, economic, etc.) in analyzing the interactions between the components of and/or the development of smart urban governance.

To sum up, this paper highlights that smart urban governance promotes a context-focused, sociotechnical way of governing cities in the smart era. More specifically, smart urban governance sees the definition of urban issues (i.e. economic, social, and environmental) as perceived and constructed through interplays between the state, market, and/or civil society. In terms of innovations of modes of governance, smart urban governance especially explores the role of situated agents and their dedication to offering the types of place-based knowledge needed for well-intended policies. For a convincing supportive role of technological intelligence, multiple functions of ICT (i.e. informing, communicating, and analyzing and designing) are required to support governance processes and handle the perceived urban problems in an appropriate way. Finally, we explicitly acknowledge the decisive role of context in analyzing the creation, application, and impacts of smart urban governance. We therefore propose smart urban governance as a sociotechnical way of governing cities in the smart era by starting with the urban issue at stake, promoting demand-driven governance modes, and shaping technological intelligence more socially, given the specific context.

## Footnotes

1 See appendix for Supplementary Material I on detailed information of the statements.

2 See appendix for Supplementary Material II on literature review and selected smart projects.

3 https://www.aisingapore.org/.

4 https://www.aalto.fi/en/aalto-built-environment-laboratory.
